# Intrapulmonary concentrations of meropenem administered by continuous infusion in critically ill patients with nosocomial pneumonia: a randomized pharmacokinetic trial

**DOI:** 10.1186/s13054-020-2763-4

**Published:** 2020-02-17

**Authors:** Adela Benítez-Cano, Sonia Luque, Luisa Sorlí, Jesús Carazo, Isabel Ramos, Nuria Campillo, Víctor Curull, Albert Sánchez-Font, Carles Vilaplana, Juan P. Horcajada, Ramón Adalia, Silvia Bermejo, Enric Samsó, William Hope, Santiago Grau

**Affiliations:** 1 Department of Anaesthesiology and Surgical Intensive Care, Hospital del Mar, Parc de Salut Mar, Barcelona, Spain; 2 Infectious Pathology and Antimicrobials Research Group (IPAR), Institut Hospital del Mar d’Investigacions Mèdiques (IMIM), Barcelona, Spain; 3 Pharmacy Department, Hospital del Mar, Parc de Salut Mar, Barcelona, Spain; 4 Department of Molecular and Clinical Pharmacology, Antimicrobial Pharmacodynamics and Therapeutics, University of Liverpool and Royal Liverpool Broadgreen University Hospital Trust, Liverpool, UK; 5 Infectious Diseases Department, Hospital del Mar, Parc de Salut Mar, Barcelona, Spain; 6 CEXS-Universitat Pompeu Fabra, Barcelona, Spain; 7 Universitat de Barcelona, Barcelona, Spain; 8 Respiratory Medicine Department, Hospital del Mar-IMIM (Hospital del Mar Research Institute) and CIBER de Enfermedades Respiratorias (CIBERES), ISCIII, Barcelona, Spain; 9 Laboratory Department, Laboratori de Referència de Catalunya, Barcelona, Spain; 10 Universitat de Barcelona, Barcelona, Spain; 11 Universitat Pompeu Fabra, Barcelona, Spain

**Keywords:** Meropenem, Population pharmacokinetics, Pharmacodynamics, Critically ill patients, Nosocomial pneumonia, Lung penetration, Dose selection

## Abstract

**Background:**

Optimal antimicrobial drug exposure in the lung is required for successful treatment outcomes for nosocomial pneumonia. Little is known about the intrapulmonary pharmacokinetics (PK) of meropenem when administered by continuous infusion (CI). The aim of this study was to evaluate the PK of two dosages of meropenem (3 g vs 6 g/day by CI) in the plasma and epithelial lining fluid (ELF) in critically ill patients with nosocomial pneumonia.

**Methods:**

Thirty-one patients (81% male, median (IQR) age 72 (22) years) were enrolled in a prospective, randomized, clinical trial. Sixteen patients received 1 g/8 h and 15 2 g/8 h by CI (8 h infusion). Plasma and ELF meropenem concentrations were modeled using a population methodology, and Monte Carlo simulations were performed to estimate the probability of attaining (PTA) a free ELF concentration of 50% of time above MIC (50% *f*T>MIC), which results in logarithmic killing and the suppression of resistance in experimental models of pneumonia.

**Results:**

The median (IQR) of meropenem AUC_0–24 h_ in the plasma and ELF was 287.6 (190.2) and 84.1 (78.8) mg h/L in the 1 g/8 h group vs 448.1 (231.8) and 163.0 (201.8) mg h/L in the 2 g/8 h group, respectively. The penetration ratio was approximately 30% and was comparable between the dosage groups. In the Monte Carlo simulations, only the highest approved dose of meropenem of 2 g/8 h by CI allowed to achieve an optimal PTA for all isolates with a MIC < 4 mg/L.

**Conclusions:**

An increase in the dose of meropenem administered by CI achieved a higher exposure in the plasma and ELF. The use of the highest licensed dose of 6 g/day may be necessary to achieve an optimal coverage in ELF for all susceptible isolates (MIC ≤ 2 mg/L) in patients with conserved renal function. An alternative therapy should be considered when the presence of microorganisms with a MIC greater than 2 mg/L is suspected.

**Trial registration:**

The trial was registered in the European Union Drug Regulating Authorities Clinical Trials Database (EudraCT-no. 2016-002796-10). Registered on 27 December 2016.

## Key points

This is the first article assessing the lung penetration of different doses of meropenem administered by continuous infusion in critically ill patients with nosocomial pneumonia. A dose increase leads to higher plasma and epithelial lining fluid concentrations allowing to achieve an optimal probability of target attainment for all susceptible microorganisms.

## Background

The relatively high incidence, rising rates of antimicrobial resistance, and suboptimal clinical outcomes of patients with nosocomial pneumonia provide the impetus to optimize the use of existing antibiotics [1–4].

Meropenem is a carbapenem antibiotic with potent activity against many of the pathogens that cause nosocomial pneumonia [1]. It exhibits time-dependent pharmacodynamics (PD); the fraction of the dosing interval that free plasma concentrations are above the MIC (*f*T>MIC) is the PK/PD index that best describes its antimicrobial efficacy [2, 3]. Although the maximal bactericidal activity for meropenem has been associated with a *f*T>MIC of 40–50% [4], a higher pharmacodynamic target of 50–100% *f*T>MIC has been suggested for critically ill patients [5]. Continuous (CI) (or extended) infusion of β-lactam antibiotics increases the *f*T>MIC and has been linked to improved clinical outcomes in some clinical studies [6–8]. The emergence of resistance is another important endpoint, so regimens must be designed to provide drug exposures that minimize the development of resistance [9, 10].

Plasma drug exposures are frequently used as a proxy for effect-site concentrations. However, in some cases, they may be misleading, and measurement of antimicrobial concentrations at the site of infection might be more relevant for predicting clinical response [11]. The epithelial lining fluid (ELF) is the most clinically relevant compartment to estimate intrapulmonary drug concentrations [12, 13]. In a murine pneumonia model for meropenem, a *f*T>MIC of 50% in ELF has been associated with logarithmic bacterial killing and suppression of resistant subpopulation amplification [14].

In this study, we investigated the utility of CI to achieve drug exposures in the human lung which are predicted to be effective and suppress the emergence of resistance. Specifically, we sought to (1) estimate plasma and ELF concentrations of meropenem in critically ill patients with nosocomial pneumonia receiving 3–6 g/day by CI, (2) describe the population pharmacokinetics (popPK) of meropenem in this population, and (3) estimate regimens that achieve effective drug exposures in ELF against Gram-negative pathogens with MIC likely to be encountered in patients with pneumonia.

## Methods

### Study design, settings, and patients

This was a single-center, prospective, open-label, randomized, comparative PK clinical trial that was conducted at a tertiary surgical intensive care unit (ICU) at the Parc de Salut Mar in Barcelona, Spain, between January 2017 and February 2019. The study was approved by the local ethics committee (Comitè Etic d’Investigació Clínica del Parc de Salut Mar; approval no. 2016/7125) and the Spanish Medicines and Health Product Agency (AEMPS; registration no. 16-0774). Furthermore, the trial was registered in the European Union Drug Regulating Authorities Clinical Trials Database (EudraCT-no. 2016-002796-10). Written informed consent was obtained from the patients or their legal representatives.

The inclusion criteria were as follows: age ≥ 18 years, diagnosis of nosocomial pneumonia and risk factors for multidrug-resistant (MDR) pathogens [*Pseudomonas aeruginosa*, *Acinetobacter* spp., and extended-spectrum β-lactamases (ESBL)-producing Gram-negative *Bacilli*] [15], and glomerular filtration rate (GFR) ≥ 50 mL/min/1.73 m^2^ (estimated using the Chronic Kidney Disease Epidemiology Collaboration (CKD-EPI) formula [16]). The exclusion criteria were allergy to β-lactams, previous use of carbapenems within 15 days, GFR < 50 mL/min/1.73 m^2^, severely impaired liver function [cirrhosis grade C by Child-Pugh classification [17]], obesity (body mass index (BMI) > 30), pregnancy, life expectancy < 3 days, and colonization [respiratory secretions or surveillance cultures (oropharyngeal and rectal swabs)] with pathogens known to be resistant to meropenem.

The diagnosis of pneumonia was based on standard clinical and laboratory criteria and defined as a new or progressive radiological pulmonary infiltrate plus two or more of the following characteristics: temperature > 38 °C or < 35 °C, leucocyte count > 11,000 or < 4000 cells/mm^3^, or purulent respiratory secretions [15].

### Data collection

The following data were collected at the onset of treatment: demographics, BMI, Charlson Comorbidity Index [18], Acute Physiology and Chronic Health Evaluation II (APACHE) score [19] and SOFA score, type of pneumonia [hospital-acquired (HAP) or ventilator-associated (VAP)], sepsis or septic shock [20], presence of fluid overload, and mechanical ventilation. HAP was defined as pneumonia not incubating at the time of hospital admission and occurring ≥ 48 h after admission. VAP was defined as pneumonia occurring > 48 h after endotracheal intubation [15]. Laboratory data such as renal function (GFR and serum urea at baseline and on day 3), biomarkers [C-reactive protein (CRP), pro-calcitonin (PCT)], serum albumin, and total serum proteins were collected.

Clinical cure at the end of treatment, length of ICU and hospital stay (LOS), 7- and 30-day all-cause mortality, in-hospital mortality, and microbiological data (isolated organism, MIC value, and microbiological eradication) were recorded. Clinical cure was defined as the resolution of signs and symptoms present at enrolment and the resolution or lack of progression of radiological signs of pneumonia during follow-up (7 to 10 days after treatment initiation) [21]. Microbiological eradication was defined as the eradication of the microorganisms cultured from respiratory samples at baseline and at the end of treatment [22]. Seven and 30-day all-cause mortality was considered as death from any cause during the 7 or 30 days following the end of treatment, and in-hospital mortality was defined as death occurring during the hospital stay. Antibiotic susceptibility testing of the isolated pathogens was determined using the Vitek2® automated system (Biomerieux, France) and interpreted according to EUCAST breakpoints (European Committee on Antimicrobial Susceptibility Testing) [23]. In a few cases, susceptibility was confirmed by E-test (Biomerieux, France). Adverse events potentially associated with meropenem were collected such as local (inflammation, pain, phlebitis or edema at the injection site), cutaneous (rash, pruritus), gastrointestinal (diarrhea, nausea/vomiting, constipation), neurological (headache, insomnia, agitation, delirium, confusion, dizziness, seizure, nervousness, paresthesia, hallucinations, somnolence), drug-induced liver injury (increased alanine aminotransferase, aspartate aminotransferase, alkaline phosphatase, lactate dehydrogenase, bilirubin), or *Clostridioides difficile*-associated diarrhea (CDAD).

### Randomization

Based on previous PK-related studies [24, 25], an initial sample size of 30 patients was considered for the desired level of significance. All included patients were randomized to receive 3 g or 6 g of i.v meropenem per day. Randomization was performed by an individual not related to the study using the SISA computer program (simple interactive statistical analysis). Randomization was balanced (1:1) without blocks or stratification. All patients received meropenem (Meropenem Accordpharma®; Accord Healthcare, S.L.U. Barcelona, Spain) at an initial loading dose (LD) of 2 g (in 50 mL of 0.9% saline infused by a central line in 15 min) followed by a CI of 3 g or 6 g/day (1 g or 2 g of meropenem over 8 h every 8 h). Meropenem by CI was diluted in 100 mL of 0.9% saline solution and injected into a central venous catheter via a volumetric pump (Braum Mesulgen, Mesulgen, Germany) with an infusion dead space of < 2 mL [26]. A maximum infusion time of 8 h was chosen based on the available meropenem stability data [27] and a stability study performed in our laboratory to confirm the previous results (data not shown). All patients received empirical combination therapy with 3 MIU/8 h of nebulized colistimethate sodium (CMS) (Accord®, Accord Healthcare, Barcelona, Spain). Nebulization was performed using a vibrating-mesh nebulizer (Aeroneb Pro®, Aerogen, Galway, Ireland) as it was described in a previous study [28].

### Pharmacokinetic study

Blood and ELF samples were obtained after the third or fourth day of treatment once a steady state had been achieved. Blood samples were collected pre-infusion and at 1.5, 3, 6, and 8 h after the start of meropenem infusion. ELF samples were obtained simultaneously at 6 h post-infusion by bronchoalveolar lavage (BAL) during a standardized fiberoptic bronchoscopy using a bronchoscopic BAL catheter procedure (Combicath®, Prodimed, Le Plessis Bouchard, France) by instilling three aliquots of sterile 0.9% saline (20 mL, 40 mL, and 40 mL). The time between the beginning of BAL and the total recovery of the three aliquots did not exceed 2 min for each, in order to minimize the free diffusion of urea through the alveolar epithelium, which might lead to falsely elevated concentrations of urea in the BAL fluid [29]. The liquid recovered from the first aliquot was rejected, since it is not considered representative of ELF [30]. Blood and ELF samples were centrifuged at 4 °C, and the supernatant was frozen at − 80 °C until analysis.

### Bioanalytical methods

Meropenem concentrations were measured using a validated high-performance liquid chromatography (HPLC) method [31] at the Pharmacy Department of Hospital del Mar. The assay was linear from 0.5 to 80 mg/L and 0.03 to 1 mg/L in the plasma and BAL, respectively. Precision and accuracy were ≤ 15% at high, medium, and low concentrations. The limit of quantification was 0.5 and 0.03 mg/L in the plasma and BAL, respectively. Normal serum saline (0.9%) was used to prepare the standard calibrators of meropenem in BAL.

Concentrations of urea in the plasma and ELF were determined with the Urea/BUN kit (Roche® professional Diagnostics, Mannheim, Germany) being the LOQ 3 mg/dL in the plasma and 0.078 mg/dL in ELF.

Meropenem concentration in ELF (MER_ELF_) was determined according to the following formula, using urea as an endogenous marker, to correct the meropenem concentrations in ELF following dilution from the BAL [32, 33]:

$$ {\mathrm{MER}}_{\mathrm{ELF}}={\mathrm{MER}}_{\mathrm{BAL}}\times {\mathrm{Urea}}_{\mathrm{SER}}/{\mathrm{UREA}}_{\mathrm{BAL}} $$

where MER_BAL_ is the meropenem concentration measured in BAL, Urea_SER_ is the urea concentration in the plasma, and Urea_BAL_ is the urea concentration in BAL.

### Population pharmacokinetic model

Population pharmacokinetic modeling was performed using the nonparametric adaptive grid (NPAG) approach embedded in Pmetrics (Los Angeles, CA, USA) [34, 35]. One-, two-, and three-compartment structural models were fitted to the data and evaluated. Elimination from the central compartment and intercompartmental distribution were modeled as first-order processes. Data were weighted using the inverse of the estimated assay variance, and additional process noise was modeled using gamma as a multiplicative error term.

Age, gender, actual body weight (ABW), APACHE score, serum creatinine, GFR, serum albumin, total serum proteins, serum urea, CRP, PCT, presence of septic shock, presence of fluid overload, and mechanical ventilation were evaluated as covariates using stepwise linear regression. Potential covariates were separately entered into the model and retained if their inclusion resulted in a statistically significant improvement in the log likelihood value and/or in the observed-predicted plots.

The fit of each model to the data was assessed using a linear regression of observed-predicted values both before and after the Bayesian step. The mean prediction error and the mean bias-adjusted squared prediction error were used to assess bias and imprecision, respectively. Models were compared by calculating twice the difference in the log likelihood values.

The final model was also evaluated graphically and statistically by visual predictive checks (VPCs) performed from normalized prediction distribution errors (NPDEs) [36]. One thousand datasets were simulated using the final population model parameters. For the VPCs, the 5th, 50th, and 95th percentiles of the simulated concentrations were processed using the R platform, plotted against elapsed time, and compared to observed concentrations. For a model in which random effects are well estimated, approximately 90% of the observed data are expected to be within the 5th to 95th prediction interval. NPDE results were summarized graphically by default as provided by the NPDE R package (version 1.2) using (i) a Q-Q plot (where Q is quantile) of the NPDE and (ii) a histogram of the NPDE.

### Other pharmacokinetic calculations

The average AUC in the plasma and ELF for each patient was estimated using the Bayesian posterior parametric estimates from the final model using the trapezoidal rule in Pmetrics. The daily average AUC (AUC_0–24_) was calculated by dividing the cumulative AUC of each patient by the total time in hours and multiplying the obtained result by 24 h. The partitioning of meropenem into ELF was described using the ratio of AUC_0–24 h_ ELF/AUC_0–24 h_ plasma.

### Monte Carlo simulations

Monte Carlo simulations (*n* = 1000) of plasma concentrations were employed to calculate the *f*T≥MIC in ELF on the third day of treatment (from 48 to 72 h post-treatment) Three different meropenem regimens (2 g of LD followed by a maintenance dose of 1 g/8 h in CI, 2 g of LD followed by a maintenance dose of 2 g/8 h in CI, and 3 g of LD followed by a maintenance dose of 3 g/8 h in CI) against a range of MIC values (0.002–16 mg/L) were examined. Human protein binding of 2% in the plasma was used to estimate free drug concentrations in the plasma [37] while measured total concentrations in ELF were regarded as equivalent to the free fraction because protein binding is expected to be negligible [38]. A probability of target attainment (PTA) ≥ 90% was considered optimal. The AUC_48–72 h_ in the plasma and ELF with two different dosages of meropenem were also simulated.

The potential toxicity of the different regimens was estimated by calculating the probability of achieving the threshold meropenem concentrations in the plasma associated with 50% risk of developing a neurotoxicity event, which has been defined as a minimum concentration (*C*_min_) in the plasma ≥ 64.2 mg/L [39].

### Statistical analysis

Dichotomous variables were compared using the chi-square test and Fisher’s exact test. Quantitative data were expressed as medians [interquartile range (IQR)] and compared using the Mann-Whitney *U* test. Correlations were analyzed using Spearman’s correlation. A *P* value of < 0.05 was considered statistically significant. The SPSS (SPSS, Chicago, IL, USA) version 24.0 statistical package was used throughout.

## Results

### Enrolment and characteristics of study patients

**Fig. 1:**
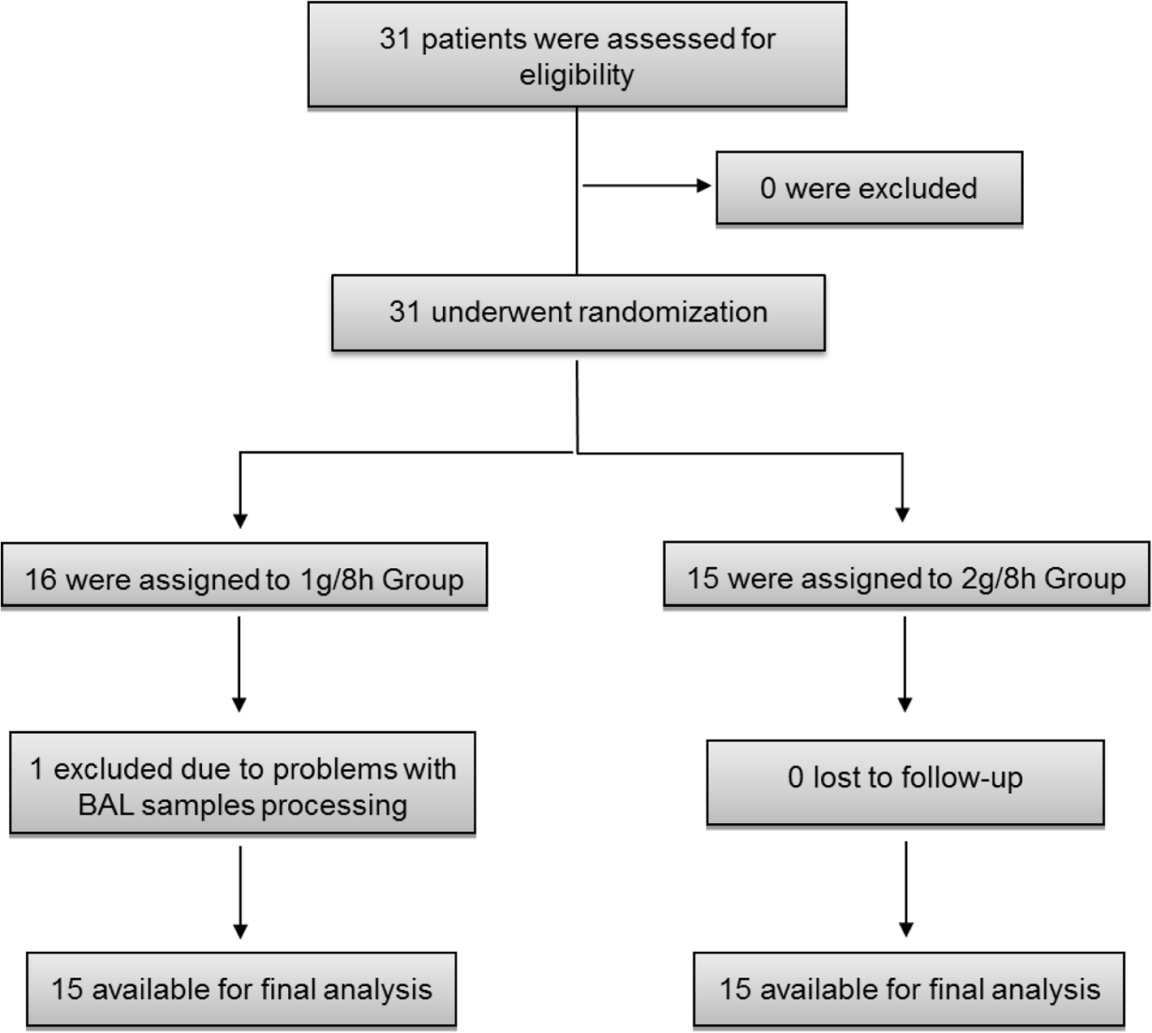
Enrolment and follow-up of the study patients

Thirty-one patients were enrolled: 16 in the 1 g/8 h group and 15 in the 2 g/8 h group. A subject in the 1 g/8 h group was excluded due to problems with BAL sample processing. To compensate, an additional patient was recruited and randomized (Fig. 1). The demographic and clinical characteristics of both groups are summarized in Table 1. Most patients were male (81%), with a median (IQR) age of 72 (22) years and APACHE score of 15 (8). No significant differences in any demographic or clinical variable were observed between the two groups except for a higher Charlson score in the 2 g/8 h group.

**Table 1:** Patient’s characteristics and clinical data comparing the two study groups

Characteristics	1 g/8 h group (*N* = 16)	2 g/8 h group (*N* = 15)	*P* value
Demographic and clinical data			
Male, *n* (%)	12 (75)	13 (86.7)	0.654
Age (years)	64.0 (20.0)	75.0 (14.0)	0.202
Body weight (kg)	74.0 (14.8)	70.0 (15.0)	0.495
BMI (kg/m^2^)	26.8 (3.8)	25.5 (6.4)	0.338
Charlson Comorbidity Index	4.0 (3.5)	6.0 (4.0)	**0.033**
APACHE II score^a^	13.0 (10.8)	15.0 (6.0)	0.423
Other clinical data^b^			
Type of pneumonia, *n* (%)			> 0.999
Ventilator-associated pneumonia	4 (25)	4 (26.7)	
Hospital-acquired pneumonia	12 (75)	11 (73.3)	
SOFA score	5 (4)	5 (5)	0.830
Septic shock, *n* (%)	4 (25)	3 (20)	> 0.999
Sepsis, *n* (%)	10 (62.5)	12 (80)	0.433
Concomitant bacteremia, *n* (%)	3 (18.8)	2 (13.3)	> 0.999
Fluid overload, *n* (%)	2 (12.5)	6 (40)	0.113
Mechanical ventilation, *n* (%)	4 (25)	4 (26.7)	> 0.999
GFR (mL/min/1.73 m^2^)	103.0 (33.0)	85.0 (30.0)	0.281
GFR < 90 mL/min/1.73 m^2^, *n* (%)	6 (37.5)	9 (60.0)	0.210
GFR > 120 mL/min/1.73 m^2^, *n* (%)	2 (12.5)	3 (13.3)	> 0.999
C-reactive protein (mg/dL)	15.0 (26.9)	17.5 (14.1)	0.654
Procalcitonin (ng/mL)	0.8 (2.1)	0.9 (3.6)	0.375
Total serum protein (g/dL)	5.2 (1.4)	5.1 (1.0)	0.830
Serum albumin (g/dL)	2.9 (0.9)	2.8 (0.6)	0.599
Clinical and microbiological outcomes			
Clinical cure, *n* (%)	13 (81.3)	11 (73.3)	0.685
Length of ICU stay (days)	19.9 (15.8)	18.2 (17.4)	0.730
Length of hospital stay (days)	39.5 (54.8)	28.0 (55.0)	0.682
Microbiological eradication, *n* (%)	4 (25.0%)	7 (46.7%)	0.494
Seven-day all-cause mortality, *n* (%)	0 (0)	0 (0)	> 0.999
Thirty-day all-cause mortality, *n* (%)	1 (6.3)	1 (6.7)	> 0.999
In-hospital mortality, *n* (%)	1 (6.3)	1 (6.7)	> 0.999
Microbiological data: specie, *n* (%)/MIC (mg/L)^c^			
Gram-negative bacteria	6 (37.5)	7 (46.7)	0.605
MDR Gram-negative bacteria	3 (18.8)	2 (13.3)	> 0.999
*Pseudomonas aeruginosa* (PA)	3 (18.8)	2 (13.3)	> 0.999
Multi-susceptible PA	2 (12.5)/≤ 2	0 (0)	0.484
MDR PA	0 (0)	1 (6.7)/16	0.484
XDR PA	1 (6.3)/8	1 (6.7)/32	> 0.999
Enterobacteriaceae	3 (18.8)	3 (20.0)	> 0.999
ESBL producers	3 (18.8) / ≤2	0 (0)	0.226
Other Gram-negative bacteria	1 (6.3)/≤ 2	3 (20.0)/≤2	0.333

*MDR* multidrug-resistant, *XDR* extensively drug-resistant, *ESBL* extended-spectrum beta-lactamases

^a^Calculated at the beginning of ICU admission

^b^Data at the onset of treatment

^c^Based on EUCAST breakpoints

In total, 25 pathogens (20 Gram-negative, 4 Gram-positive, and 1 virus) were isolated in respiratory cultures from 17 patients (9 in the 1 g/8 h group and 16 in the 2 g/8 h group). In 14 (45%) patients, no pathogen was isolated. Five patients had a polymicrobial infection. All patients with Gram-positive and viral isolates were in the 2 g/8 h group (2 methicillin-susceptible and 2 methicillin-resistant *Staphylococcus aureus* and 1 influenza B virus).

More patients in the 2 g/8 h group had a positive culture in BAL fluid (10/15 (66.7%) compared to 5/16 (31.3%) in the 1 g/8 h group (*P* = 0.049). Five patients had positive blood cultures with Gram-negative bacteria being 3 in the 1 g/8 h group and 2 in the 2 g/8 h group with no differences between the groups (*P* > 0.999). Only those patients with Gram-negative isolates were included in the PK/PD analysis. The Gram-negative isolates were 3 *Haemophilus influenzae*, 6 Enterobacteriaceae (2 susceptible *Escherichia coli* and 2 ESBL-*E. coli*, 1 *Klebsiella pneumoniae*, and 1 ESBL-*K. pneumoniae*), and 5 *P. aeruginosa* (2 multi-susceptible, 1 MDR, and 2 extensively drug-resistant *P. aeruginosa*). Microbiological data and distributions of MICs in the two groups are shown in Table 1. All included patients initially received an empirical combination therapy of intravenous meropenem plus 3 MIU/8 h of nebulized colistimethate sodium due to the high prevalence of MDR *P. aeruginosa* in our unit. After having the results of the microbiological cultures, meropenem was finally used empirically in 18 patients (58%) and as targeted therapy in 13 (42%) patients. In those cases with a MDR or XDR *P. aeruginosa* isolation, the initial combination therapy of meropenem plus nebulized CMS was maintained and monotherapy with meropenem was used only when a more susceptible Gram-negative bacteria were isolated. When a Gram-negative bacterium resistant to meropenem was cultured (2 patients in the 2 g/8 h group and 1 patient in the 1 g/8 h group), escalation was done being ceftolozano-tazobactam plus CMS the most frequent combination.

In seven patients clinical cure was not achieved (three in the 1 g/8 h group and four in the 2 g/8 h group), but only two of them died, one in each group. In three patients, the initial treatment with meropenem was changed by ceftolozane-tazobactam with late favorable clinical response (one in the 1 g/8 h group and two in the 2 g/8 h group). In two patients, the clinical cure was achieved after a long treatment of meropenem plus nebulized CMS plus linezolid (one of each group). Finally, the other two died, one in the 1 g/8 h group due to a stroke on the eighth day of treatment with no documented clinical cure at that point and the other in the high dose group due to MRSA pneumonia.

No adverse events related to meropenem treatment were observed in any patient. The BAL procedure was well tolerated in all cases.

### Pharmacokinetic data

**Fig. 2:**
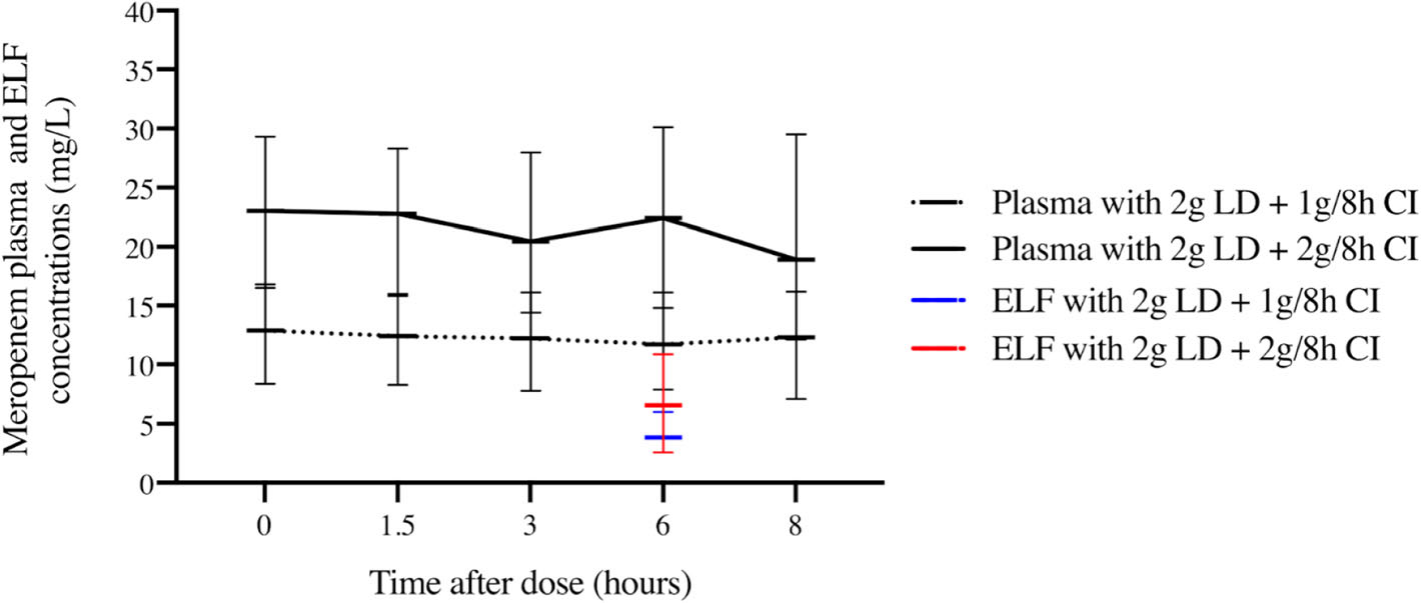
Meropenem plasma and ELF concentration-time profiles. Meropenem plasma and ELF concentration time profiles of patients receiving a loading dose of 2000 mg i.v followed by a maintenance dose of 1 g q8h and 2 g q8h i.v. Intensive sampling was performed after the second day of treatment. A statistically significant difference was observed in plasma concentrations at any time points between the two groups (meropenem plasma concentrations of 12.9 (8.4) vs 23.0 (12.8) at pre-dose; 13.4 (7.3) vs 23.9 (16.7) at 1.5 h; 13.3 (8.3) vs 21.4 (15.5) at 3 h; 11.7 (8.2) vs 22.4 (15.3) at 6 h, and 12.3 (9.1) vs 18.9 (17.4) at 8 h, in the 1 g/8 h and 2 g/8 h groups, respectively).

The concentration-time profiles of meropenem in the plasma and ELF in both groups are shown in Fig. 2. Median (IQR) meropenem plasma concentrations in the 2 g/8 h group were statistically higher at all times points compared to the 1 g/8 h group. In ELF, concentrations were also higher in the 2 g/8 h group, but this difference did not reach statistical significance (6.6 (8.3) mg/L vs 3.9 (3.2) mg/L), respectively (*P* = 0.102). A high interindividual variability was observed in ELF exposure within both groups.

### Population pharmacokinetic model

A total of 151 meropenem plasma concentrations and 30 ELF concentrations were included in the population analysis. A 3-compartment linear model, with zero-order input and first-order clearance from the central compartment, best described the data. Concentrations of meropenem in ELF were modeled by assuming ELF was a homogenous compartment with volume, *V*_ELF_. Compartments were connected by first-order intercompartmental rate constants.

Despite different covariates, such as BMI, serum creatinine, and GFR, having a relationship with the estimated clearance, they were not included in the final model because they did not improve the goodness-of-fit. Estimates for central tendency, dispersion, and 95% credibility limits for the population PK parameters are shown in Table 2.

**Table 2:** Population pharmacokinetic parameters of meropenem

Parameter (units)	Median	Mean	95% credibility limits	Standard deviation
CL (L/h)	11.219	12.464	8.539–15.589	5.570
*V* (L)	10.143	12.500	8.385–17.194	6.929
*K*_12_ (h^−1^)	26.696	22.987	23.706–27.897	8.072
*K*_21_ (h^−1^)	7.601	11.721	3.853–21.014	9.730
*K*_13_ (h^−1^)	18.539	17.317	13.124–21.815	5.611
*K*_31_ (h^−1^)	25.614	24.451	23.575–28.602	5.892
*V*_ELF_ (L)	19.424	25.319	19.321–28.525	10.735

*CL* clearance; *V* volume of the central compartment; *K*_*12*_, *K*_*21*_, *K*_*13*_, and *K*_*31*_ first-order intercompartmental rate constants; *V*_ELF_ volume of the ELF compartment

**Fig. 3:**
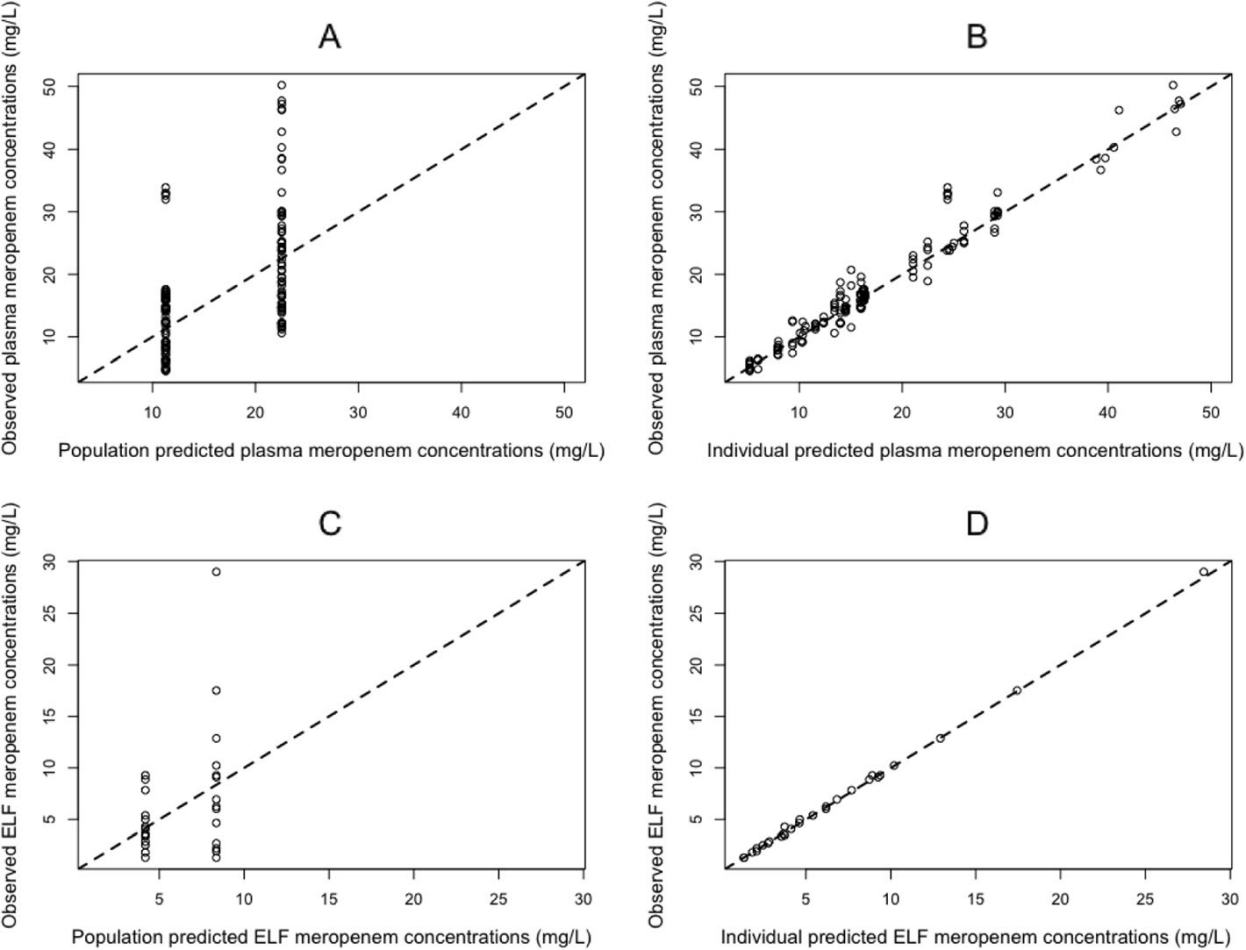
Population and individual predicted meropenem concentrations vs observed meropenem concentrations in the plasma and in ELF. Population (**a**) and individual (**b**) predicted meropenem concentrations vs observed concentrations of meropenem in the plasma (**a** and **b**, respectively) and in ELF (**c** and **d**, respectively). The broken line is the line of identity (observed = predicted concentrations)

The observed-predicted values for meropenem concentrations in the plasma and ELF before and after the Bayesian step are shown in Fig. 3. After maximum a posteriori probability (MAP)-Bayesian estimation, a linear regression of the observed-vs-predicted values in the plasma had an intercept and slope of 0.0159 (CI95 − 0.685–0.717) and 1.02 (CI95 0.984–1.05), respectively, and an *R*^2^ = 0.956. The bias and imprecision were both acceptable (bias = − 0.0803 mg/L and imprecision 1.23 mg/L). For ELF, the observed-vs-predicted plot had an intercept and slope of − 0.0784 (CI95 − 0.211–0.0546) and 1.00 (CI95 0.988–1.02), respectively, and an *R*^2^ = 0.999. The bias and imprecision were both acceptable (bias = 0.114 mg/liter and imprecision 0.189 mg/L).

Visual predictive check plots of the different doses (1 g/8 h and 2 g/8 h) in the plasma and ELF based on 1000 simulations with the final model are given in Fig. 7. The normal distribution of normalized prediction distribution errors (NPDEs) in the plasma and ELF confirmed the adequacy of the model for dosing simulations. Median (IQR) average values of AUC_0–24_ in the plasma and ELF estimated using the Bayesian posterior parametric estimates for each patient are shown in Table 3. Both AUC_0–24_, in the plasma and ELF, were statistically higher in the 2 g/8 h group than in the 1 g/8 h group. The penetration ratio was similar between the groups.

**Table 3:** Median (IQR) average AUC_48–72 h_ in the plasma and ELF in the two dose groups estimated using the Bayesian posterior parametric estimates for each patient

	1 g/8 h group (*N* = 16)	2 g/8 h group (*N* = 15)	*P*
AUC_0–24_ in the plasma (mg h/L)	322.7 (225.6)	492.3 (354.1)	0.004
AUC_0–24_ in ELF (mg h/L)	101.5 (78.7)	175.9 (258.7)	0.047
Ratio AUC_ELF_/AUC_plasma_ (%)	31.8 (33.9)	36.4 (44.4)	> 0.999

**Fig. 4:**
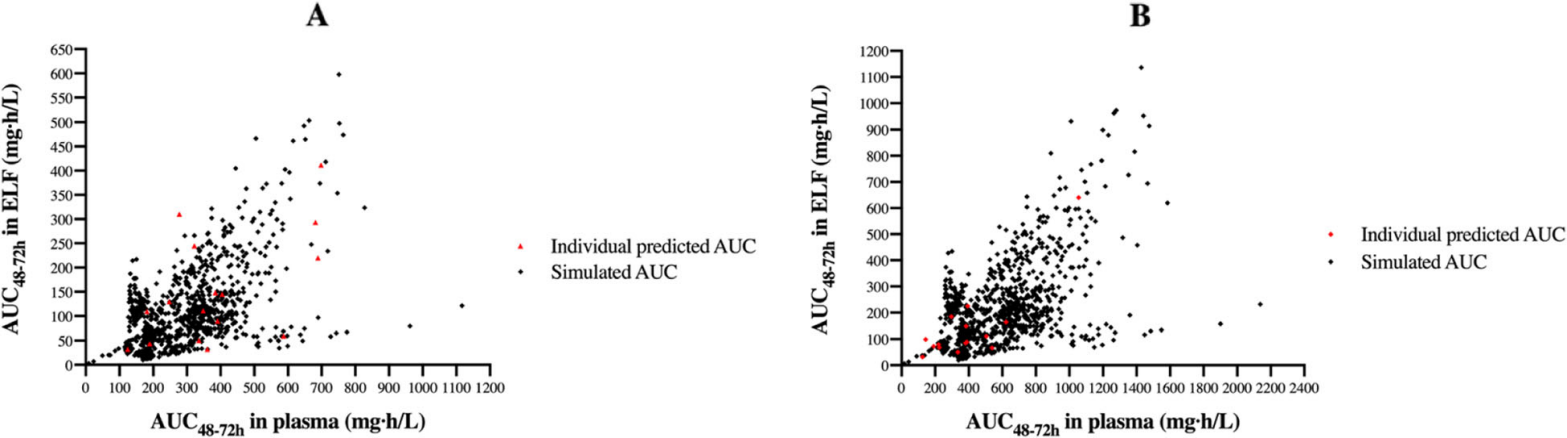
Comparison of the individual predicted AUC_48–72 h_ and the simulated AUC_48–72 h_ in the plasma and ELF. Comparison of the individual predicted AUC_48–72 h_ using the Bayesian posterior estimated concentrations (red diamonds) and the simulated AUC_48–72 h_ (black diamonds) in the plasma and ELF with two different dosages of meropenem (2 g loading dose (LD) followed by 1 g/8 h as a continuous infusion (**a**) and 2 g LD followed by 2 g/8 h as a continuous infusion (**b**))

The AUC_0–24_ in ELF showed a moderate positive linear correlation with AUC_0–24_ in the plasma, age, and APACHE score (Spearman rho = 0.533, 0.575, and 0.537, respectively) (*P* < 0.05) and an inverse correlation with patients’ ABW, BMI, and GFR (Spearman rho = − 0.688, − 0.598, and − 0.376, *P* < 0.05). Figure 4 shows the comparison between the individual predicted AUC_48–72 h_ using the Bayesian posteriors (red diamonds) and the simulated AUC_48–72 h_ (black diamonds) in the plasma and ELF with two different dosages of meropenem.

### Probability of target attainment

**Fig. 5:**
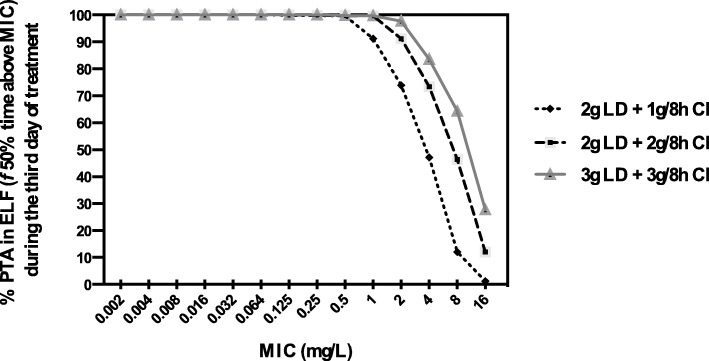
Probability of target attainment (PTA) in ELF of different dosages regimens. PTA in ELF of different dosages of meropenem: 2 g loading dose (LD) followed by 1 g/8 h, 2 g LD followed by 2 g/8 h, and 3 g LD followed by 3 g/8 h; administered as a continuous infusion during the third day of treatment (from 48 to 72 h after the start of the treatment)

**Fig. 6:**
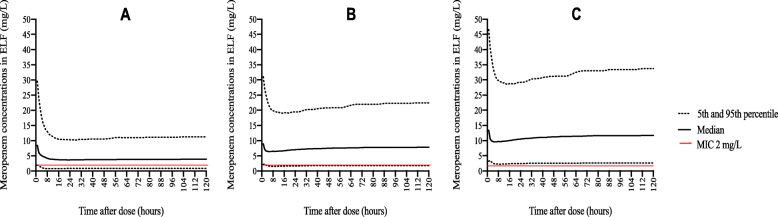
Comparison of the time course of meropenem concentrations simulated in ELF. Comparison of the median, percentile 5th and 95th time course of meropenem concentrations simulated in ELF during 4 days with different dosing regimens of meropenem as a continuous infusion (2 g loading dose (LD) followed by 1 g/8 h (**a**), 2 g LD followed by 2 g/8 h (**b**), and 3 g LD followed by 3 g/8 h (**c**))

The probability of target attainment (PTA) for achieving 50% *f*T>MIC in ELF for the three different meropenem doses on day 3 of treatment is shown in Fig. 5. With the lowest dose (2 g LD + 1 g/8 h by CI), an optimal PTA could be achieved for isolates with MICs of < 2 mg/L. With the administration of a double maintenance dose (2 g LD + 2 g/8 h), a PTA ≥ 90% in ELF could be attained for isolates with MIC up to 2 mg/L, which is the current susceptibility breakpoint [23]. A dosage increases to 3 g LD + 3 g/8 h by CI did not result in significantly greater coverage of MIC. We also estimated the dose needed for isolates with intermediate susceptibility (MIC between 2 and ≤ 8 mg/L) that was estimated to be as high as 8 g/8 h, which is four times higher than the maximum licensed meropenem dose. Figure 6 shows the simulated meropenem concentration-time profiles in ELF of each tested regimen.

**Fig. 7:**
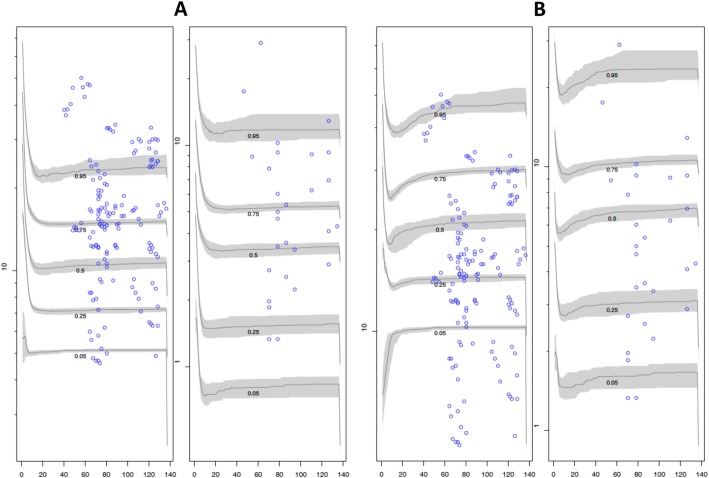
Visual predictive check plot for meropenem concentrations. Visual predictive check plot for meropenem concentrations at a dose of 2 g loading dose + 1 g/8 h (**a**) and at a dose of 2 g loading dose + 2 g/8 h (**b**) in the plasma and ELF (left and right, respectively). Observed concentrations (blue circles); simulated concentrations at the designated quantile given by the number on the line (lines)

Similar results were obtained with the administration of meropenem in an extended infusion of 4 h. The probability of target attainment (PTA) for achieving 50% *f*T>MIC in ELF was also assessed on day 3 of treatment for three different meropenem doses (1 g/8 h, 2 g/8 h, and 3 g/8 h) administered by extended infusion (4 h) (Fig. 7). With the lowest dose of 1 g/8 h, an optimal PTA could be achieved for isolates with MICs of < 2 mg/L, and with higher doses (2 g/8 h and 3 g/8 h), the coverage increased to a MIC up to 2 mg/L.

From the point of view of toxicity, the probability of achieving a *C*_min_ in the plasma of meropenem ≥ 64.2 mg/L during the first 3 days of treatment was estimated to be 0% for the two lowest doses and 1.7% for the 3 g/8 h dose. In comparison, the use of the highest dose (8 g/8 h) resulted in nearly half of the patients (49.0%) achieving this potentially toxic trough concentration.

### PK/PD in ELF and clinical outcomes

Twelve patients with documented Gram-negative bacterial infections were eligible for the PK-PD sub-study.

All patients that achieved clinical cure had a *f*T>MIC > 50% in ELF in both groups, compared to patients who failed treatment (*f*T>MIC 33.3%, *P* = 0.045). Regarding the microbiological results, a higher proportion of patients with eradication achieved an optimal ELF target, but this difference was not statistically significant (88.9% vs 66.7%, *P* = 0.455). No correlation was found between the duration of mechanical ventilation (days) and meropenem ELF or plasma meropenem concentrations (data not shown).

## Discussion

Meropenem is a licensed agent for the treatment of nosocomial pneumonia [15]. As for other β-lactams, the pharmacodynamics of meropenem is optimized with the use of prolonged infusions, especially CI [6–8]. In recent years, higher meropenem dosages are being recommended to avoid suboptimal exposure [40], but the clinical benefits are still unknown.

Several studies have identified the administration of carbapenems as an independent risk factor for the emergence of carbapenem-resistant Gram-negative bacteria in ICU patients [41, 42]. In this scenario, one proposed strategy to minimize the emergence of resistance of meropenem is the administration to achieve sufficient drug exposures to kill both susceptible and prevent the emergence of resistant subpopulations [43, 44]. Tam et al. reported that selective amplification of subpopulations of *P. aeruginosa* with reduced susceptibilities to meropenem was suppressed with a *C*_min_/MIC of ≥ 6.2 in the plasma [43]. In fact, some authors consider that no single agent can achieve an adequate exposure to provide a cell kill sufficient to allow optimal clinical outcomes and simultaneously suppress amplification of less susceptible subpopulations of organisms in infections caused by some bacteria, such as *P. aeruginosa* [44].

In pneumonia, a PK/PD target at the site of the infection of *f*T>MIC of 50% in ELF has been associated with bacterial killing and suppression of resistant subpopulation amplification in a murine model of pneumonia [14]. Unlike other PK/PD targets calculated in the plasma [45, 46], this has been more precisely defined at the site of the infection. This was the pharmacodynamic target used in this analysis and is higher than that often cited for the efficacy of the carbapenem class.

Several studies have assessed the PK/PD of meropenem in ELF [10, 47–49], but only two of them have focused on critically ill patients [10, 48]. Both studies assessed ELF meropenem concentration and lung penetration with the use of both intermittent and 3-h extended infusion. Although a higher penetration ratio (AUC_ELF_/AUC_plasma_) of meropenem was observed with the use of an extended infusion, none of the two regimens achieved an optimal PK/PD target in ELF [48].

To our knowledge, this is the first population PK study of meropenem in critically ill patients with nosocomial pneumonia which compared two different dosages administered as a CI. Our results showed that the administration of 2 g/8 h of meropenem as a CI allowed to achieve higher meropenem concentrations in the plasma and ELF compared to the 1 g/8 h dose, although a high interindividual variability in the meropenem concentrations in ELF was observed. However, even the administration of the highest licensed dose (2 g/8 h) of meropenem by CI in patients with conserved renal function did not result in an optimal ELF target attainment for a substantial fraction of the population.

The median penetration ratio into the lungs was approximately 30% and was comparable between both groups. Similar penetration ratios (AUC_ELF_/AUC_plasma_) have been reported in other contexts [10, 14, 48]. Lodise et al. simulated a penetration of 26% in patients with VAP after the administration of a single dose of 2 g meropenem given over 3 h [10], and the authors also reported a substantial variability in the lung penetration ratio (10th and 90th percentiles of 3.7% and 178.0%, respectively). In the same way, the PROMESSE study performed in 55 critically ill patients with severe pneumonia treated with 1 g/8 h reported a statistically higher AUC penetration ratio in the extended infusion group (3 h) compared to the intermittent group [mean (SD) 29 (± 3) % vs 20 (± 3) % (*P* = 0.047)] [48]. In our study, all ELF samples were obtained at the same time; hence, a precise estimate of the concentration-time profile of meropenem in ELF was not possible.

In our study, AUC in ELF was positively correlated with AUC in the plasma, suggesting that plasma exposures are a potential surrogate marker of lung exposures. However, the correlation was not especially strong, and plasma concentrations cannot be used to confidently predict lung concentrations. Although we acknowledge that routine measurement of drug levels in ELF is infeasible in all critically ill patients with pneumonia, direct lung measurement in those patients at risk of treatment failure (especially if pathogens with high MIC values are suspected) may be reasonable and should be considered.

In our study, a higher proportion of patients with clinical cure achieved an optimal PK/PD ratio at the infection site compared to those who failed. Although our findings suggest an association between the achievement of an optimal PK/PD of meropenem in lungs and better clinical outcomes in patients with Gram-negative respiratory infections, this was not an objective in our study. In addition, all patients received concomitant treatment with nebulized CMS, which could have influenced patients’ clinical outcomes, especially on microbiological eradication. Due to all these reasons, our results have to be confirmed in a larger prospective clinical study.

Our work is the first study assessing the achievement of a PK/PD target at the site of infection with the use of different dosages of meropenem administered by CI. Our results suggest that an optimal PTA can be achieved for MIC values < 2 mg/L with a dose of 1 g/8 h and for MIC values < 4 mg/L with a higher dose of 2 g/8 h. A lower coverage (a lower MIC dilution) was reported with the use of the same doses of meropenem administered by extended infusion (over 3 h) in the PROMESSE study. The authors used a similar target, 54% *f*T>MIC in ELF, a value associated with microbiological response in a clinical study of Li et al. [50], and observed that an optimal PTA could only be attained for MIC breakpoints of 0.5 mg/L and 1 mg/L with meropenem doses of 1 g/8 h and 2 g/8 h, respectively, administered by extended infusion (3 h). Similarly, Drusano et al. confirmed that even with the highest licensed meropenem dose, the 50% *f*T>MIC in ELF target could not be achieved even at very low MIC values (0.25 mg/L) [14]. Although the administration of meropenem by CI seems to improve drug exposure in ELF compared with extended infusion [48], it might not be sufficient to cover all intermediate Gram-negative pathogens causing nosocomial pneumonia in critically ill patients with conserved renal function. In those cases, alternative strategies may be required, especially when pathogens with high MIC values (> 2 mg/L) may be present. In fact, we estimate the meropenem dose by CI needed to achieve an optimal PTA for all considered intermediate strains (MIC between 2 and 8 mg/L) [23] that would have to be as high as 8 g/8 h, a dose that is four times higher than the highest approved meropenem dose, and that is related to a high probability of toxicity.

Our study has several limitations. Firstly, it is a relatively small single-center study. Secondly, all ELF measurements were performed at a single time point. The collection of ELF samples at different times would have allowed a more precise determination of the concentration-time profile in ELF. However, a recent study demonstrated that ELF models constructed with concentrations from sparse ELF sampling time points result in exposure estimates similar to those constructed from robustly sampled ELF profiles [51]. Thirdly, all samples were collected on the same day, so intraindividual variability during the treatment period could not be measured [52]. ELF samples were all collected in the infected lung; as distribution of inflammation is heterogeneous, the collection of samples in the clear lung could have led us to know the differences in the antibiotic diffusion. Finally, the relatively limited sample size and the use of combination therapy with nebulized CMS made difficult to correlate the PK/PD target in ELF with clinical and microbiological outcomes. Nevertheless, this study provides important and useful information about the meropenem dosages that should be used in clinical practice for treating nosocomial pneumonia caused by Gram-negative bacteria in critically ill patients, considering both the achievement of clinical cure and possibly the prevention of the emergence of resistance.

## Conclusions

In conclusion, the administration of meropenem by continuous infusion improves drug exposure in the ELF, but the use of the highest licensed dose (2 g/8 h) is still needed to achieve a target attainment in ELF of > 90% for isolates with an MIC up to 2 mg/L in patients with conserved renal function. Alternative therapeutic strategies may be required for the treatment of nosocomial pneumonia caused by Gram-negative bacteria in critically ill patients when MDR strains with high MIC values are suspected.

## Data Availability

The datasets used and analyzed during the current study are available from the corresponding author on reasonable request.

## Abbreviations

ABW: Actual body weight
APACHE: Acute Physiology and Chronic Health Evaluation
BAL: Bronchoalveolar lavage
BMI: Body mass index
CDAD: *Clostridioides difficile*-associated diarrhea
CI: Continuous infusion
CI95: Confidence interval of 95%
CKD-EPI: Chronic Kidney Disease Epidemiology Collaboration
*C*_min_: Minimum concentration
CMS: Colistimethate sodium
CRP: C-reactive protein
ELF: Epithelial lining fluid
ESBL: Extended-spectrum beta-lactamases
EUCAST: European Committee on Antimicrobial Susceptibility Testing
*f*T>MIC: Percentage of time remaining concentration above MIC
GFR: Glomerular filtration rate
HAP: Hospital-acquired pneumonia
HPLC: High-performance liquid chromatography
ICU: Intensive care unit
IQR: Interquartile range
LD: Loading dose
LOS: Length of hospital stay
MDR: Multidrug-resistant
MER_BAL_: Meropenem concentration in BAL
MER_ELF_: Meropenem concentration in ELF
MIC: Minimal inhibitory concentration
PCT: Pro-calcitonin
PD: Pharmacodynamic
PK: Pharmacokinetic
popPK: Population pharmacokinetic
PTA: Probability of target attainment
SD: Standard deviation
SOFA: Sequential Organ Failure Assessment
Urea_BAL_: Urea concentration in BAL
Urea_SER_: Urea concentration in the plasma
VAP: Ventilator-associated pneumonia
XDR: Extensively drug-resistant
